# Comprehensive aroma profiles and the underlying molecular mechanisms in six grape varieties with different flavors

**DOI:** 10.3389/fpls.2025.1544593

**Published:** 2025-04-28

**Authors:** Guang Wu, Yuchen Xin, Ruihua Ren, Huawei Chen, Bowei Yang, Maosheng Ge, Sha Xie

**Affiliations:** ^1^ College of Enology, Northwest A & F University, Yangling, China; ^2^ College of Water Resources and Architectural Engineering, Northwest A & F University, Yangling, China

**Keywords:** grape, aroma, volatiles, HS-SPME/GC-MS, RNA-seq

## Abstract

Aroma is a critical factor in determining grape quality, develops through complex interactions among various volatile compounds. This study revealed the differences of the six grape varieties with three different aroma types though the HS-SPME/GC-MS and RNA-sequencing technologies. Muscat-type grapes ('Shine 13' and 'Shine Muscat') exhibited the highest monoterpene and C13-norisoprenoid level, correlating with elevated expression of *DXS, TPS, and CCD4b* genes in the MEP/MVA pathways. Strawberry-type cultivars (particularly 'Hutai 8') accumulated abundant esters linked to high *AAT* expression, while neutral aromatic varieties showed enriched C6/C9 compounds associated with upregulated *LOXA* and *ADH2*. Muscat-type grapes dominated monoterpenes with OAVs >1, which explained the abundant Muscat flavors, while neutral aromatic aroma cultivars had the most abundant C6/C9 compounds OAVs associated with leaf-like scents. Strawberry-type cultivars exhibited the highest esters OAVs with strawberry aroma profiles. WGCNA analysis revealed four specific modules correlated with aroma compound biosynthesis correlated with alcohols (88genes), carbonyl compounds (451genes), fatty acids (110 genes), and monoterpenes (790genes) accumulation in these grapes, respectively. These findings were expected to advance our understanding of the metabolic pathways responsible for grape aroma and could provide valuable recommendations for the enhancement of grape aromatic quality.

## 1 Introduction

Grapes are one of the most famous fruits, typically consumed either as table grapes or processed into juice and wine (Dong et al., 2023). Grape aroma, a critical aspect of grape quality, arises from intricate chemical processes involving multiple compound classes, which shape the distinct sensory traits of various grape cultivars (Ghaste et al., 2015). Previous studies have confirmed that monoterpenes, C6/C9 compounds, C13-norisoprenoids, and esters are the dominant substances contributing to grape aromas (Wu et al., 2020; Feng et al., 2022; Wang W.-N. et al., 2023). Grapes are categorized into Muscat, strawberry, and neutral aromatic cultivars based on the level of monoterpenes. Among these compounds, monoterpenes are the primary aroma compounds to Muscat cultivars (Mateo and Jiménez, 2000), C6/C9 compounds are regarded as the fundamental, underlying scents in grapes (Wu et al., 2016); Yao et al. (2021) found that esters were the key volatile compounds in ‘Hutai 8’ (Wu et al., 2016). In grape berries, aroma compounds occur in free and glycosidically bound forms. Glycosidically bound aromas predominantly exist in glycosylated molecules that are hydrophilic, non-volatile, and flavorless. Free volatile aroma can be converted into glycosylated precursor by UDP-glycosyltransferases (UGTs) (Ghaste et al., 2015), which can directly provide grapes with floral, fruity, rose-like, and green flavors (Wang et al., 2021; Liu et al., 2022). However, until now little information about comprehensive and detailed free and bound aroma profiles of different aroma types of grape varieties has been reported.

In grape berry, the synthesis of aromatic substances included terpene metabolism (**Supplementary Figure S1A**), amino acid metabolism (**Supplementary Figure S1B**), and fatty acid metabolism (**Supplementary Figure S1C**). Monoterpenes, including α-terpineol, linalool, and geraniol, are considered the primary fragrant substances in Muscat grape cultivars (Luan et al., 2006). Variation in Muscat flavor among grape cultivars is attributed to the diverse monoterpene profiles (Liu et al., 2022). Monoterpenes and norisoprenoids are primarily synthesized through the methyl-erythritol phosphate (MEP) and mevalonate (MVA) pathways (Li et al., 2025). The MEP pathway takes place in plastids, whereas the MVA pathway occurs in the cytosol (Vranová et al., 2013). Terpene biosynthesis begins with the generation of isopentenyl pyrophosphate (IPP), followed by the isomerization to form dimethylallyl pyrophosphate (DMAPP) (Leng et al., 2023). The genesis of grape aroma compounds also stems from the metabolism of fatty acids, whether saturated or unsaturated, which are channeled through β-oxidation and the LOX-HPL metabolic pathway, yielding alcohols, aldehydes, ketones, acids, and esters (Kiralan et al., 2019). Moreover, these volatiles act as signaling molecules for pollination and defense against pathogen invasion (D’Auria et al., 2007). It has been indicated that alcohol acyltransferase (AAT) plays a key role in catalyzing the formation of volatile esters in fruits by linking alcohols with acyl-CoA (Beekwilder et al., 2004). Two principal types of amino acids exist, classified by their structure: aliphatic and aromatic amino acids. Leucine, isoleucine, valine, alanine, and cysteine are among the amino acids that can serve as precursors for volatile compounds in fruits, which can metabolize into alcohols, aldehydes, ketones, and esters in plants (Pan et al., 2012). However, at present, the pathway and molecular regulation mechanism of grape aroma synthesis have not been fully clear.

In this research, the neutral grape cultivars ‘Red Globe’ (*V. vinifera*) and ‘Moldova’ (a hybrid of *V. labrusca* and *V. vinifera*), alongside the Muscat-scented ‘Shine Muscat’ and ‘Shine 13’ (both hybrid of *V. labrusca* and *V. vinifera*), as well as the strawberry-aroma varieties ‘Summer Black’ and ‘Hutai 8’ (both hybrids of *V. labrusca* and *V. vinifera*) were selected to analyze distinct aroma compounds and identify candidate genes impart to these aroma differences in the different aroma-type grapes. The volatile compounds of grape berries were identified by GC−MS. Weighted gene co-expression network analysis (WGCNA) was conducted to explore the candidate genes related to the target traits. RNA sequencing and qRT-PCR methods were employed to examine transcriptomic signatures. By integrating transcriptome and metabolome, we identified the differences in the aroma profiles of different aroma types and elucidate the genetic basis of cultivar-specific aroma formation. The findings from this research offer valuable understanding regarding the differences among aroma-type grape cultivars, the fundamental biological processes involved, and the core molecular dynamics within grape berries. This not only enhances our understanding of the chemical basis of grape aroma diversity but also offers practical guidance for vineyard management and breeding strategy. Moreover, our findings open up several avenues for future research. The correlations we found between certain molecular markers and aroma traits can be further explored through genetic studies, potentially leading to the development of new grape cultivars with desired flavor characteristics.

## 2 Materials and methods

### 2.1 Grape cultivars

This research used six grape cultivars with different aroma types as experimental materials. These grape cultivars were collected at maturity from Weinan-based vineyards (34°50′N,109°48′E), Shanxi, China. The grape cultivars in this study were all grown on their own roots and protected by rain shelters, ensuring uniformity in vineyard management across all varieties. Three biological replicates were gathered for each type of grape, with each replicate consisting of 200 berries. These berries were chosen at random from a pool of at least 50 vines, sampled from both the North-South sides of the vineyard canopy to ensure representation. The collected samples were then flash-frozen using liquid nitrogen and preserved at a temperature of -80 degrees Celsius for subsequent analysis of the metabolome and transcriptome.

### 2.2 Extraction of free and glycosidically bound aroma compounds

Forty frozen grape berries were de-seeded, and the pulp and skin were mashed into a powder with 2.0g of polyvinylpyrrolidone (PVPP) and 0.5g of *D*-gluconolactone, using liquid nitrogen. The pulp was processed by crushing, blending, and a 4-hour soak at 4°C. Following this, it was processed by centrifugation at 8000g for 15 minutes to yield purified grape juice. At last, clear grape juice was used to detect the free volatile compounds. For each respective sample, three independent extractions were conducted.

The methods of extracting the glycosidically bound aroma compounds were consistent with (Wen et al., 2015). This process was repeated three times for each sample to create independent extracts.

### 2.3 HS-SPME/GC-MS analysis of aroma compounds

An automated HS-SPME system using a 2-cm DVB/CAR/PDMS fiber measuring 50/30 μm (Supelco, Bellefonte, PA, USA) and a CTC Combi PAL autosampler (CTC Analytics, Zwingen, Switzerland) was employed for extraction aromatic compounds from clear juice or mixtures. The SPME fiber underwent an activation step was consistent with (Lan et al., 2016).

Volatile compound analysis of six different grape cultivars was performed operating with an Agilent 6890 gas chromatograph integrated with an Agilent 5975 detector (GC-MS, Agilent Technologies, Santa Clara, CA). The conditions of GC-MS were consistent with (Sun et al., 2020).

The aroma compounds were pinpointed by correlating their mass spectral fingerprints with entries in the NIST14 mass spectrometry database and by aligning retention indices of the compounds against known reference values. This process was streamlined through the Automated Mass Spectral Deconvolution and Identification System (AMDIS), which seamlessly computed the retention indices and mass spectral data.

### 2.4 Odor activity values

OAVs were determined applying the formula OAV = c/t, with c representing the concentration of free volatiles and t referring to the odor threshold, derived from literature data.

### 2.5 RNA- sequencing and quantitative real-time PCR analyses

The Vazyme FastPure Universal Plant Total RNA Isolation Kit (RC411) was utilized to isolate total RNA from the pulp and skin of six grape varieties. RNA samples were used to build sequencing libraries, and subsequently examined using the BGISEQ-500 platform (Beijing Genomic Institution, www.genomics.org.cn) to produce 150-base end-sequences. Subsequently, a thorough transcriptomic analysis was conducted using the filtered initial reads sequencing data to the *Vitis vinifera* template genome accessible at Ensembl Plants. Gene expression was quantified using the FPKM approach. The ‘limma’ package in R (v. 4. 2. 3) was utilized to analyze expression Expression differences. A threshold of absolute log (2Fold change) ≥1 and *P* < 0.05 was applied for identifying differentially expressed genes (DEGs). The DEGs were employed in functional category enrichment within the KEGG pathway framework.

The primers for qRT-PCR were detailed in **Supplementary Table S2**.

### 2.6 Statistical analysis

Monoterpene concentrations of compound were compared using one-way ANOVA to evaluate differences. Subsequently, a Tukey’s HSD test was conducted with a significance level of *P* < 0.05, utilizing the SPSS 20.0 software (IBM, Armonk, NY). Histograms were generated using OriginPro 2021 (OriginLab Corporation, Northampton, MA). ‘pheatmap’ package in R (v. 4. 2. 3) was used to plot the heatmaps. The KEGG analysis was performed using KOBAS, a freely accessible online data analysis service (Bu et al., 2021). The ‘ggplot2’ package in R (v. 4.2.3) was used to plot the KEGG analysis enrichment pathway and KOG function classification histograms. Co-expression analysis was conducted utilizing gene expression data across the six grape cultivars.

## 3 Results and discussion

### 3.1 Free aroma compounds in six grape varieties

**Figure 1:**
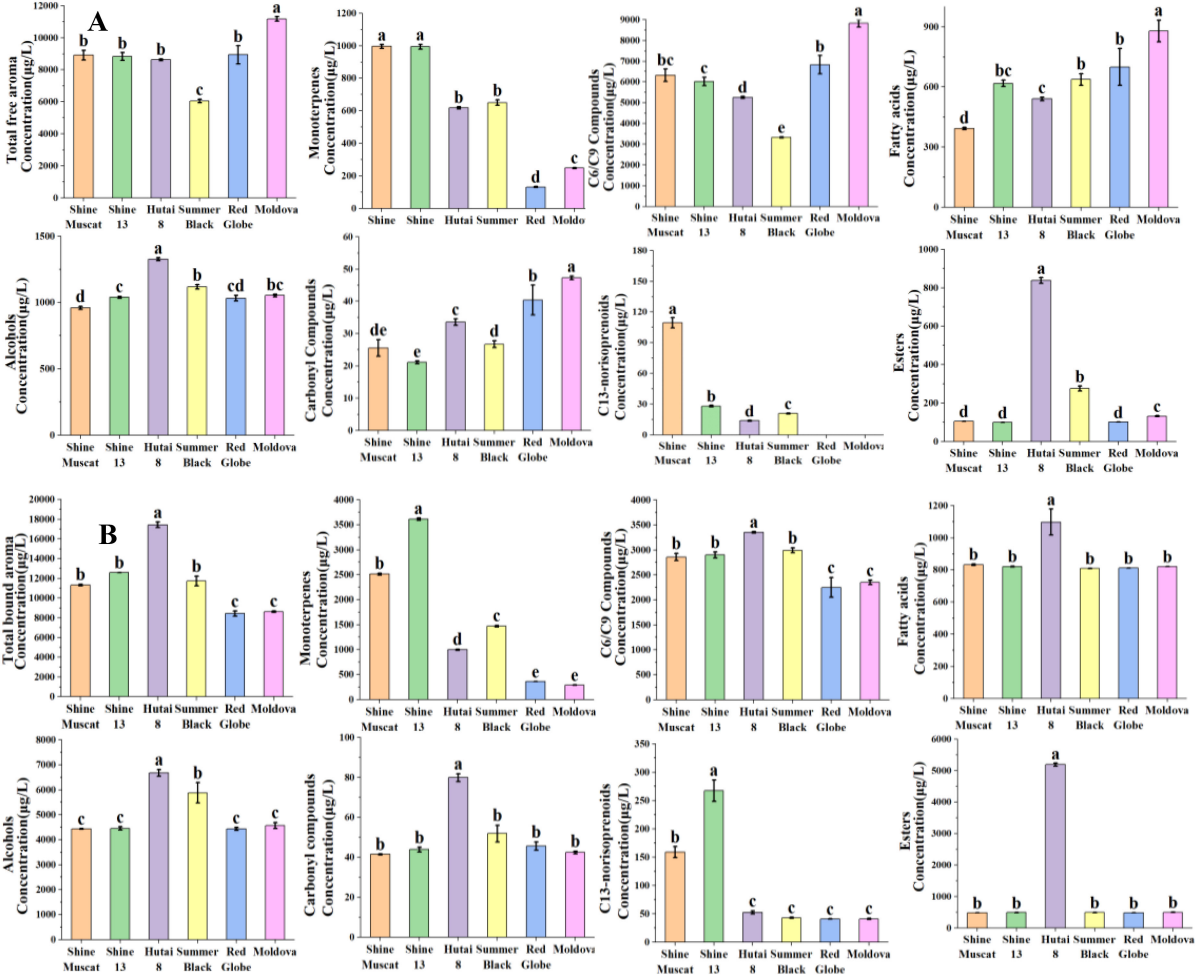
Concentrations of free **(A)** and **(B)** aroma compounds in six grape cultivars. Different letters in each graph indicated significant differences at *P* < 0.05.

Aromatic substances reside in the grape exocarp and pulp in free and bound glycosides. The free volatiles directly impart the characteristic flavors to the grapes (Wu et al., 2019). In this research, a total of 61, 61, 57, 56, 41, and 48 free aroma compounds were found in ‘Shine Muscat’, ‘Shine 13’, ‘Hutai 8’, ‘Summer Black’, ‘Red Globe’ and ‘Moldova’, individually (**Supplementary Table S2**; **Figure 1A**). The free aroma volatiles concentration was highest for ‘Moldova’ (11814.15μg/L) and ‘Red Globe’ (8935.6μg/L), followed by ‘Shine Muscat’ (8912.43μg/L) and ‘Shine 13’ (8829.95μg/L), while it was lowest concentration for ‘Hutai 8’ (8626μg/L) and ‘Summer Black’ (6056.3μg/L) (**Supplementary Table S2**; **Figure 1A**). Of the grape cultivars included in this study, ‘Shine Muscat’ and ‘Shine 13’ among the six table grapes as Muscat varieties (Wang et al., 2021). ‘Hutai 8’ and ‘Summer Black’ were classified as strawberry-type cultivars (Wu et al., 2019).”Red Globe” and “Moldova” were categorized as neutral aromatic cultivars (Xiang et al., 2022). ‘Shine Muscat’ and ‘Shine 13’ exhibited the highest levels of free monoterpenes, with 996.23μg/L and 994.03μg/L, respectively (**Supplementary Table S2**; **Figure 1A**). Monoterpenes are considered to be the dominant compound of the Muscat cultivars (Mateo and Jiménez, 2000). In this study, linalool, *trans*-*β*-ocimene, *cis*-*β*-ocimene, *β*-myrcene, and geraniol were the main monoterpenes in Muscat-type cultivars. Among them, linalool was the richest monoterpene in Muscat-type cultivars. Fenoll et al. (2009) investigated composition, concentration, and aromatic impact of monoterpenes during the development of muscat humburg grape, identifying linalool as the most significant contributor to the aroma profiles. In line with our results, earlier research has indicated that linalool, geraniol, and *β*-myrcene were the dominant monoterpene in Muscat-type cultivars (Wang H. et al., 2023; Wang W.-N. et al., 2023; Yue et al., 2022). The concentration of free C13-norisoprenoids was highest in ‘Shine Muscat’(109.38μg/L) and ‘Shine 13’(28.09μg/L), followed by ‘Summer Black’(20.85μg/L) and ‘Hutai 8’(13.78μg/L), while in neutral aromatic cultivars were not detected. 6-Methyl-5-hepten-2-ol was the most abundant C13-norisoprenoids in Muscat-type cultivars (**Supplementary Table S2**; **Figure 1A**). Volatiles esters have been identified as the predominant volatile compounds in some hybrid cultivars derived from *V. vinifera and V. labrusca*, significantly contributing to the overall aromatic profile of these hybrids (Yang et al., 2011; Wu et al., 2016). In this study, the concentration of free esters was highest in ‘Hutai 8’(838.16μg/L) and ‘Summer Black’(275.55μg/L). Furthermore, ethyl 3-hydroxybutyrate was the most abundant ester in ‘Hutai 8’ and ‘Summer Black’, followed by ethyl butanoate and ethyl hexanoate (**Supplementary Table S2**; **Figure 1A**). Similarly, Yao et al. (2021) reported that the aldehydes and esters were the predominant aroma compounds in ‘Hutai 8’ grape. In accordance with our findings, previous studies reported ethyl butanoate and ethyl hexanoate were rich in strawberry-type grape cultivars (Qian et al., 2019; Yang et al., 2009). The highest levels of free C6/C9 compounds were found in ‘Moldova’ (8821.79μg/L) and ‘Red Globe’ (6828.49μg/L). Moreover, (E)-2-hexenal were the most abundant C6/C9 compounds in ‘Moldova’ and ‘Red Globe’, followed by hexanal, (E)-2-hexenol, and hexanoic acid (**Supplementary Table S2**; **Figure 1A**). Consistent with our results, previous investigations reported the neutral aromatic cultivars had the highest (E)-2-hexenal, followed by hexanal (Aubert and Chalot, 2018; Wang W.-N. et al., 2023). Terpenes and esters are present in low concentration in neutral cultivars, Koyama et al. (2022) observed that C6 compounds account for the main aroma compounds, though the concentration of terpenes and esters tends to be low in neutral grapes. It should be recognized that the concentration of C6/C9 compounds was the highest aroma compound in six grape cultivars. Wu et al. (2016) have indicated that the C6 compounds are the basic background volatile aromas, which is consistent with our findings. However, the aroma characteristics of grape berries mainly depend on the contents of monoterpenes and esters. Additionally, the neutral aromatic cultivars also had the most abundant carbonyl compounds. Among them, the concentration of benzaldehyde was the highest compound in neutral aromatic grapes.

### 3.2 Glycosidically-bound aroma compounds in six grape varieties

The bound aroma is mainly present in hydrophilic, non-volatile, and flavorless glycosylated molecules and the bound aroma compounds can be converted into their free form through hydrolysis (Wu et al., 2020). In grapes, the concentration of glycosylated aroma acting as a key indicator of the aromatic potential (Hernandez-Orte et al., 2015). In this research, a total of 59, 59, 55, 53, 48, and 46 bound aroma compounds were identified in ‘Shine Muscat’, ‘Shine 13’, ‘Hutai 8’, ‘Summer Black’, ‘Red Globe’, ‘Moldova’, respectively (**Supplementary Table S2**; **Figure 1B**). The bound monoterpene concentration was highest in ‘Shine 13’ (3613.85μg/L) and ‘Shine Muscat’(2512.65μg/L), succeeded by ‘Summer Black’ (1462.62μg/L) and ‘Hutai 8’ (996.78μg/L), while the neutral aromatic cultivars ‘Red Globe’ (359.66μg/L) and ‘Moldova’ (290.28μg/L) had the lowest bound monoterpene concentration. This result was consistent with the concentration of free monoterpene. This study revealed that nerol was the highest concentration bound monoterpene identified in ‘Shine 13’ and ‘Shine Muscat’, followed by linalool and geraniol. Corresponding to our results, previous studies reported that bound nerol, linalool, and geraniol were the major glycosidically monoterpenes in Muscat-type cultivars (Ruiz-García et al., 2014). Linalool and nerol can be synthesized by geraniol, are characterized by their distinctive floral, sweet, and fruit aroma, and serve as the primary compounds responsible for the aromatic profile of Muscat grapes (Fenoll et al., 2009). The abundant linalool, nerol, and geraniol may suggest the related biosynthesis pathways in Muscat grapes. Matsumoto and Ikoma (2016) found that linalool, nerol, and geraniol are the most primary aromatic compounds and responsible for the aroma in Muscat cultivars. The bound C13-norisoprenoids concentration was highest in ‘Shine 13’ (267.64μg/L) and ‘Shine Muscat’ (158.99μg/L), followed by ‘Hutai 8’ (52.63μg/L) and ‘Summer Black’ (42.97μg/L), however, ‘Red Globe’ (40.9μg/L) and ‘Moldova’ (41.07μg/L) had the lowest C13-norisoprenoids. This result was in line with the free C13-norisoprenoids, and the bound C13-norisoprenoids were higher than the free form. 6-Methyl-5-hepten-2-ol was the most abundant bound C13-norisoprenoid in Muscat-type cultivars, while strawberry and neutral cultivars were not identified. The concentration of bound esters was highest for strawberry-type cultivars, specifically rich in ‘Hutai 8’ (5190.29μg/L). Ethyl 3-hydroxybutyrate was the predominant ester in ‘Hutai 8’, which is consistent with its high concentration among the free esters. Fatty acids undergo metabolic processes within fruits to yield volatile esters (Qin et al., 2014). Therefore, alcohols, as metabolic precursors, may play important roles in influencing the compounds and distribution of volatile esters within grape berries (Qian et al., 2019). The concentration of bound alcohols was found to be the highest in ‘Hutai 8’ (6674.97μg/L) and ‘Summer Black’ (5877.63μg/L). Phenylethyl alcohol emerged as the most abundant alcohol, followed by benzyl alcohol, which in agreement with an earlier study on strawberry-type cultivars (Qian et al., 2019).

### 3.3 Principal component analysis of free and glycosidically-bound aroma compounds in six grape cultivars

**Figure 2:**
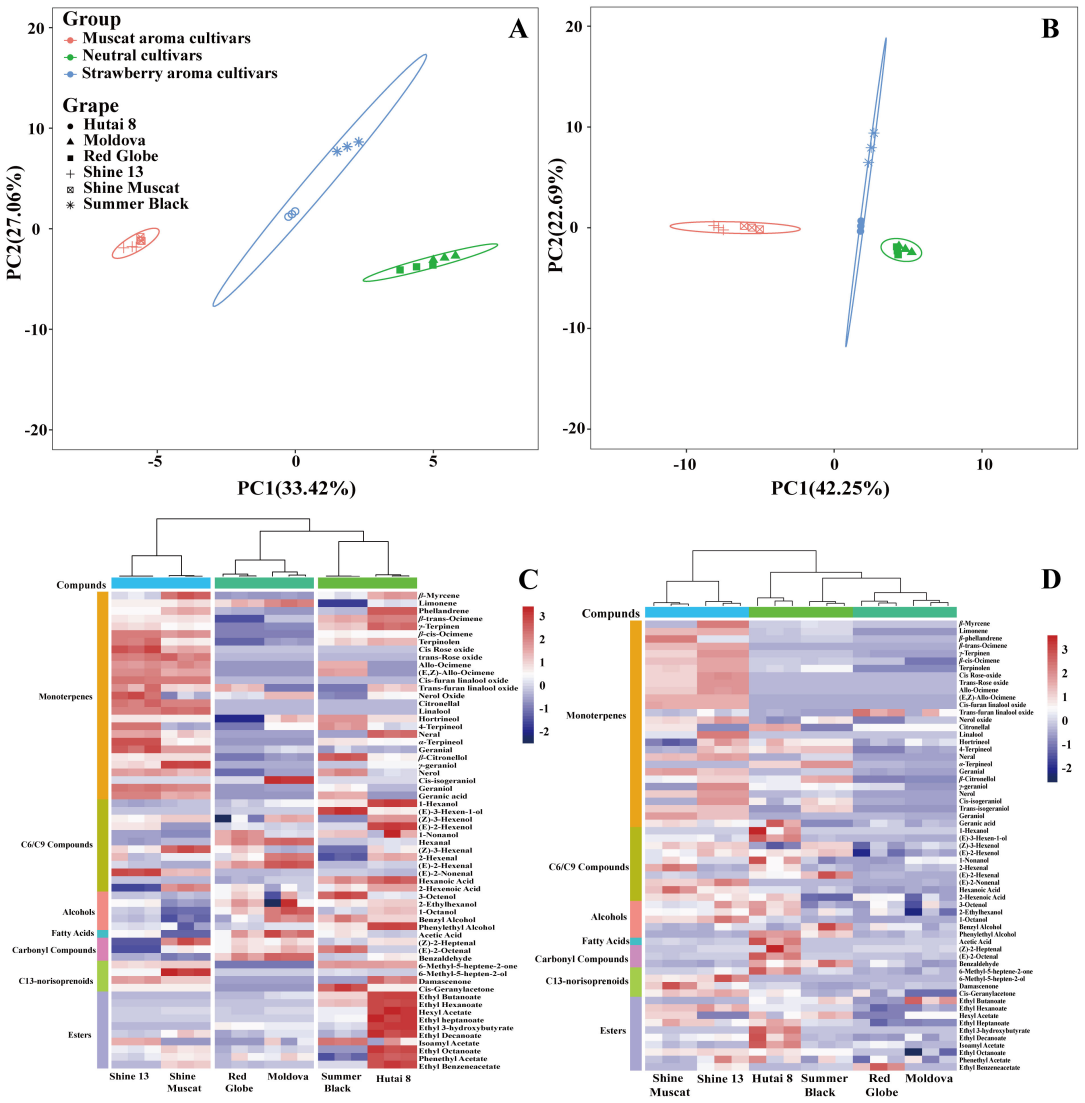
Principal component analysis (PCA) of free **(A)** and bound **(B)** volatile compounds of six grape cultivars. Heatmap analysis of free **(C)** and bound **(D)** aroma compounds.

PCA was conducted on the six grape cultivars using the free and bound aroma compounds as the variables to evaluate their cultivar characteristics. The first and second principal components (PC1 and PC2) accounted for 33.42% and 27.06% of the total variance in the free aroma compounds of the six grape cultivars (**Figure 2A**), as well as 42.25% and 22.69% of the total variance in the bound aroma compounds of the six grape cultivars (**Figure 2B**), respectively. It was observed that both the free and glycosidically-bound aromas in the six grape cultivars were divided into three parts: ‘Shine Muscat’ and ‘Shine 13’, ‘Hutai 8’ and ‘Summer Black’, as well as ‘Moldova’ and ‘Red Globe’ were in the same group, respectively. Accordingly, all grapes in the same cultivar type had similar aroma profiles.

### 3.4 Heatmap analysis of free aroma compounds

To further explore the variations of the free and bound aroma profiles of six grape cultivars, a heatmap analysis was performed. The results revealed significant inter-cultivar differences in both the abundance and diversity of aroma compounds (**Figure 2C**), corroborating previous findings that even cultivars sharing the same aroma classification exhibit distinct compound profiles (Wu et al., 2019). Hierarchical clustering grouped the cultivars into three distinct clusters: Muscat-flavored cultivars (‘Shine Muscat’ and ‘Shine 13’); strawberry-aroma cultivars (‘Hutai 8’ and ‘Summer Black’); neutral-aroma cultivars (‘Red Globe’ and ‘Moldova’). This observation was in alignment with the findings presented in Section 3.3, suggesting that grape cultivars of the same aroma type share similar aroma profiles and genetic backgrounds. In this study, a total of 27 monoterpenes, 12 C6/C9 compounds, 5 alcohols, 1 fatty acid, 3 carbonyl compounds, C13-norisoprenoids, and 10 esters were detected. Muscat-flavored clusters exhibited strong positive correlations with monoterpenes and C13-norisoprenoids. Strawberry-aroma clusters were predominantly associated with alcohol and esters. Neutral-aroma clusters showed higher affinity for C6/C9 compound. The distinct clustering further may support the utility of volatile compound profiling as a tool for cultivar classification and quality prediction.

### 3.5 Heatmap analysis of glycosidically-bound aroma compounds

**Figure 2D** illustrated the variability of bound aroma compounds across six grape cultivars. A total of 58 bound volatiles were identified, comprising 28 monoterpenes, 10 C6/C9 compounds, 5 alcohols, 1 fatty acid, 3 carbonyl compounds, 2 C13-norisoprenoids, and 10 esters. Hierarchical clustering revealed two distinct groups: Muscat-flavored cultivars (‘Shine Muscat’ and ‘Shine 13’); neutral-aroma cultivars (‘Red Globe’ and ‘Moldova’). This clustering aligns with their free aroma profiles, suggesting conserved biosynthetic regulation of both free and bound forms within cultivars. However, ‘Hutai 8’ and ‘Summer Black’ (strawberry-aroma cultivars) were not clustered, suggesting the lower concentration of bound aroma compounds in ‘Summer Black’. Notably, Muscat-type cultivars (‘Shine 13’ and ‘Shine Muscat’) exhibited strong positive correlations with bound monoterpene and C13-norisoprenoids, mirroring their free volatile associations. In contrast, strawberry-aroma cultivars (‘Hutai 8’ and ‘Summer Black’) showed higher affinity for fatty acid, carbonyl compounds, and ester, potentially contributing to their characteristic ripe-fruit aroma. These findings highlight the parallel between bound and free volatile profiles.

### 3.6 OAVs of volatile aroma compounds in six grape varieties

This study described the results of the OAVs in six grape cultivars, which were employed to evaluate the impact of each component on grape aromas (**Table 1**). The OAV, derived from the concentration of the aroma compound over its odor detection threshold, serves as a crucial metric for assessing the quality of grape aromas (Yang et al., 2019). However, only the OAV > 1 of volatile compounds was regarded as contributing to the aroma (Genovese et al., 2013b).

**Table 1:** OAVs of free volatile aroma compounds from six grape varieties.

Aroma compunds(μg/L)	‘Shine Muscat’	‘Shine 13’	‘Hutai 8’	‘Summer Black’	‘Red Globe’	‘Moldova’	odour threshold (μg/L)	Odour descriptor
Monoterpenes								
Numbers	9	9	5	4	2	2		
Sub total	98.86 ± 0.34b	110.95 ± 3.95a	9.87 ± 0.06c	7.28 ± 0.06c	6.72 ± 0.38c	7.93 ± 0.31c		Rose, Flora
C6/C9 Compounds								
1-Hexanol	<1	<1	<1	<1	<1	<1	8000^1^	Resin, Flower, Green^1^
(E)-3-Hexen-1-ol	<1	<1	<1	<1	<1	<1	1000^2^	Moss, Fresh^2^
(Z)-3-Hexenol	3.69 ± 0.17ab	3.13 ± 0.11b	4.26 ± 0.07a	1.79 ± 0.01c	1.52 ± 0.14c	3.97 ± 0.11ab	40^1^	Grass^1^
(E)-2-Hexenol	2.99 ± 0.17d	15.92 ± 0.54b	35.89 ± 0.83a	9.46 ± 0c	9.83 ± 0.24c	8.7 ± 0.53c	40^3^	Green, Leaf, Walnut^3^
1-Nonanol	<1	<1	<1	<1	<1	<1	50^4^	Rose-Orange^4^
Hexanal	240.8 ± 7.25bc	218.43 ± 7.59c	102.78 ± 5.37d	80.1 ± 2.71d	257.2 ± 2.93b	310.2 ± 4.69a	10^5^	Green, Grassy^5^
(Z)-3-Hexenal	133.93 ± 9.11a	66.19 ± 4.04b	65.27 ± 14.77b	ND	74.87 ± 10.88b	32.62 ± 3.05c	0.25^4^	Grass^4^
2-Hexenal	<1	<1	<1	<1	<1	<1	400^6^	Herbaceous, Green^6^
(E)-2-Hexenal	180.83 ± 11.9b	150.06 ± 5.14c	87.41 ± 3.9d	69.84 ± 2.54d	190.66 ± 22.16b	267.92 ± 5.29a	17^3^	Green, Apple-Like^3^ Bitter Almond-Like
(E)-2-Nonenal	33.1 ± 0.78b	59.75 ± 1.95a	5.05 ± 0.28d	8 ± 0.53c	4.61 ± 0.24d	5.1 ± 0.15d	0.69^5^	Fatty, Green^5^
Hexanoic acid	<1	<1	1.34 ± 0.03a	1.16 ± 0.07b	<1	<1	420^1^	Sweat^1^
2-Hexenoic acid	<1	<1	<1	<1	<1	<1	1000^4^	Fatty, Rancid^4^
Numbers	6	6	7	6	7	6		
Subtotal	595.55 ± 28ab	513.65 ± 19.38c	302.32 ± 12.63d	170.44 ± 0.79e	538.91 ± 36.34bc	628.88 ± 7.74a		
Alcohols								
3-Octenol	3.22 ± 0.23b	3.46 ± 0.11b	4.1 ± 0.08b	9.00 ± 0.6a	5.29 ± 0.7b	3.81 ± 0.01b	1^7^	Cucumber, Earth, Fat, Floral, Mushroom^7^
2-Ethylhexanol	<1	<1	<1	<1	<1	<1	270^8^	Rose, Green^8^
1-Octanol	<1	<1	<1	<1	<1	<1	10000^9^	Chemical, Metal, Burnt^9^
Benzyl alcohol	<1	<1	<1	<1	<1	<1	10000^10^	Sweet, Flower^10^
Phenylethyl Alcohol	<1	<1	<1	<1	<1	<1	14000^11^	Rose^11^
Numbers	1	1	1	1	1	1		
Subtotal	3.22 ± 0.23b	3.46 ± 0.11b	4.10 ± 0.08b	9.00 ± 0.6a	5.29 ± 0.7b	3.81 ± 0.01b		
Fatty acids								
Acetic acid	<1	<1	<1	<1	<1	<1	180000^5^	Vinegar-like^5^
Numbers	0	0	0	0	0	0		
Subtotal	<1	<1	<1	<1	<1	<1		
Carbonyl Compounds								
(Z)-2-Heptenal	<1	<1	<1	<1	<1	<1	13^12^	Green^12^
(E)-2-Octenal	1.08 ± 0.03c	<1	1.03 ± 0d	1.22 ± 0.02a	1.09 ± 0.03bc	1.13 ± 0.02b	3^10^	Green Leaf, Walnut^10^
Benzaldehyde	<1	<1	<1	<1	<1	<1	350^10^	Almond^10^
Numbers	1	1	1	1	1	1		
Subtotal	1.08 ± 0.03c	<1	1.03 ± 0d	1.22 ± 0.02a	1.09 ± 0.03bc	1.13 ± 0.02b		Grass
C_13_-norisoprenoids								
6-Methyl-5-heptene-2-one	<1	<1	<1	<1	ND	ND	50^10^	Green, Citrus-Like^10^
6-Methyl-5-hepten-2-ol	1.97 ± 0.1a	<1	ND	<1	ND	ND	50^10^	Pepper, mushroom, rubber^5^
Damascenone	51.16 ± 0.13c	66.42 ± 4.01b	78.83 ± 6.46a	ND	ND	ND	0.05^12^	Apple, Rose, Honey^12^
*Cis*-Geranylacetone	36.33 ± 0.22b	36.29 ± 0.24b	35.71 ± 0.39b	81.74 ± 6.67a	ND	ND	0.06^13^	Floral^13^
Numbers	3	2	2	1	0	0		
Sub total	89.46 ± 0.25c	103.04 ± 4.26b	114.54 ± 6.85a	81.90 ± 6.67c	ND	ND		Floral, Fruity
Esters								
Ethyl butanoate	1.38 ± 0.05d	1.56 ± 0.02d	114.26 ± 1.51a	46.55 ± 0.1b	1.44 ± 0.05d	15.02 ± 1.04c	2.4^5^	Fruity^5^
Ethyl hexanoate	<1	<1	<1	<1	<1	<1	580^12^	Fruit, Fat^12^
Hexyl acetate	<1	<1	<1	<1	<1	<1	10^14^	Fruit, Herb^14^
Ethyl heptanoate	4.78 ± 0c	4.78 ± 0c	5.05 ± 0.02a	4.82 ± 0.01b	4.78 ± 0c	4.78 ± 0c	2^4^	Wine-like, Brandy, Fruity^4^
Ethyl 3-hydroxybutyrate	<1	<1	<1	<1	<1	<1	20000^2^	Grape-like^2^
Ethyl decanoate	<1	<1	<1	<1	<1	<1	200^1^	Grape^1^
Isoamyl acetate	<1	<1	<1	<1	<1	<1	30^15^	Banana, Fruity, Pear^15^
Ethyl octanoate	1.12 ± 0b	1.12 ± 0b	1.13 ± 0a	1.12 ± 0b	1.12 ± 0b	1.12 ± 0b	14^1^	Apple, Peel, Fruit^1^
Phenethyl acetate	<1	<1	<1	<1	<1	<1	250^12^	Rose, Honey, Tobacco^12^
Numbers	3	3	3	3	3	3		
Subtotal	7.29 ± 0.05d	7.46 ± 0.02d	120.44 ± 1.49a	52.49 ± 0.1b	7.35 ± 0.06d	20.92 ± 1.04c		Fruity
Total	795.46 ± 28.9a	738.56 ± 27.72b	552.3 ± 21.11c	322.33 ± 8.24d	559.36 ± 37.51c	662.67 ± 9.12ab		

Data are presented as the mean ± standard deviation of three biological replicates; Different letters indicate significant differences at *P* < 0.05; ND, not detected; Reference, 1: (Ferreira et al., 2000); 2: (Noguerol-Pato et al., 2014); 3: (Genovese et al., 2013a); 4: (Kaya et al., 2022); 5: (Czerny et al., 2008); 6: (Genovese et al., 2013a); 7: (Yang et al., 2010); 8: (Pino and Mesa, 2006); 9: (Moyano et al., 2009); 10: (Buttery et al., 1988); 11: (Vilanova et al., 2013); 12: (Mayr et al., 2015); 13: (Guo et al., 2021); 14: (Xiao et al., 2023); 15: (Chang et al., 2014).

A total of 9, 9, 5, 4, 2, and 2 monoterpenes in ‘Shine Muscat’, ‘Shine 13’, ‘Hutai 8’, ‘Summer Black’, ‘Red Globe’, and ‘Moldova’ had OAVs >1, respectively. The Muscat-type cultivars exhibited the highest OAVs among the six grapes, with especially high proportions for linalool and rose oxide, which impart the floral, rose, and sweet taste characteristics (Wu et al., 2019). Corresponding to our results, previous results discovered linalool and *α*-terpineol with OAVs>1 as being linked to the Muscat aroma (Yang et al., 2024). It was observed that 6, 6, 7, 6, 7, and 6 free C6/C9 compounds in ‘Shine Muscat’, ‘Shine 13’, ‘Hutai 8’, ‘Summer Black’, ‘Red Globe’ and ‘Moldova’ had OAVs >1, respectively. The OAVs of C6/C9 compounds, which are known to primarily possess green leaf or floral and fruity odor (Kalua and Boss, 2009; Yue et al., 2023), were relatively high, thus may significantly influence the formation of the volatile profiles of six grape cultivars. Wu et al. (2016) reported that the C6 compounds are the basic background volatile aromas, while the aroma characteristics of grape pulp and peel mainly depend on the contents of monoterpenes and esters. Among the C6/C9 compounds, the OAV of hexanal, (Z)-3-hexenal, and (E)-2-hexenal were present in relatively high in six grape varieties. Yang et al. (2009) documented that hexanal and (E)-2-hexenal were the dominant C6 volatile compounds and they were detected in nearly all the grape samples, which were consistent with our results. A total of 3, 2, 2, 1, 0, and 0 free C13-norisoprenoids in ‘Shine Muscat’, ‘Shine 13’, ‘Hutai 8’, ‘Summer Black’, ‘Red Globe’ and ‘Moldova’ had OAVs >1, respectively. Damascenone and cis-geranylacetone were the main factors influencing the aroma profiles of both Muscat-type and strawberry-type cultivars, due to their relatively high OAVs and low thresholds. These compounds can contribute to the development of apple, rose, honey, and floral notes in these grape berries (Mayr et al., 2015; Guo et al., 2021). A total of 3, 3, 3, 3, 3, and 3 free esters in ‘Shine Muscat’, ‘Shine 13’, ‘Hutai 8’, ‘Summer Black’, ‘Red Globe’, and ‘Moldova’ had OAVs >1, respectively. However, ‘Hutai 8’ and ‘Summer Black’ exhibited the highest OAVs in esters, with ethyl butanoate as the primary compound with an OAV >1, thereby contributing to the fruity aroma profile of these grape varieties.

### 3.7 Characterization and functional profiling of genes showing differential expression in three grape varieties

**Figure 3:**
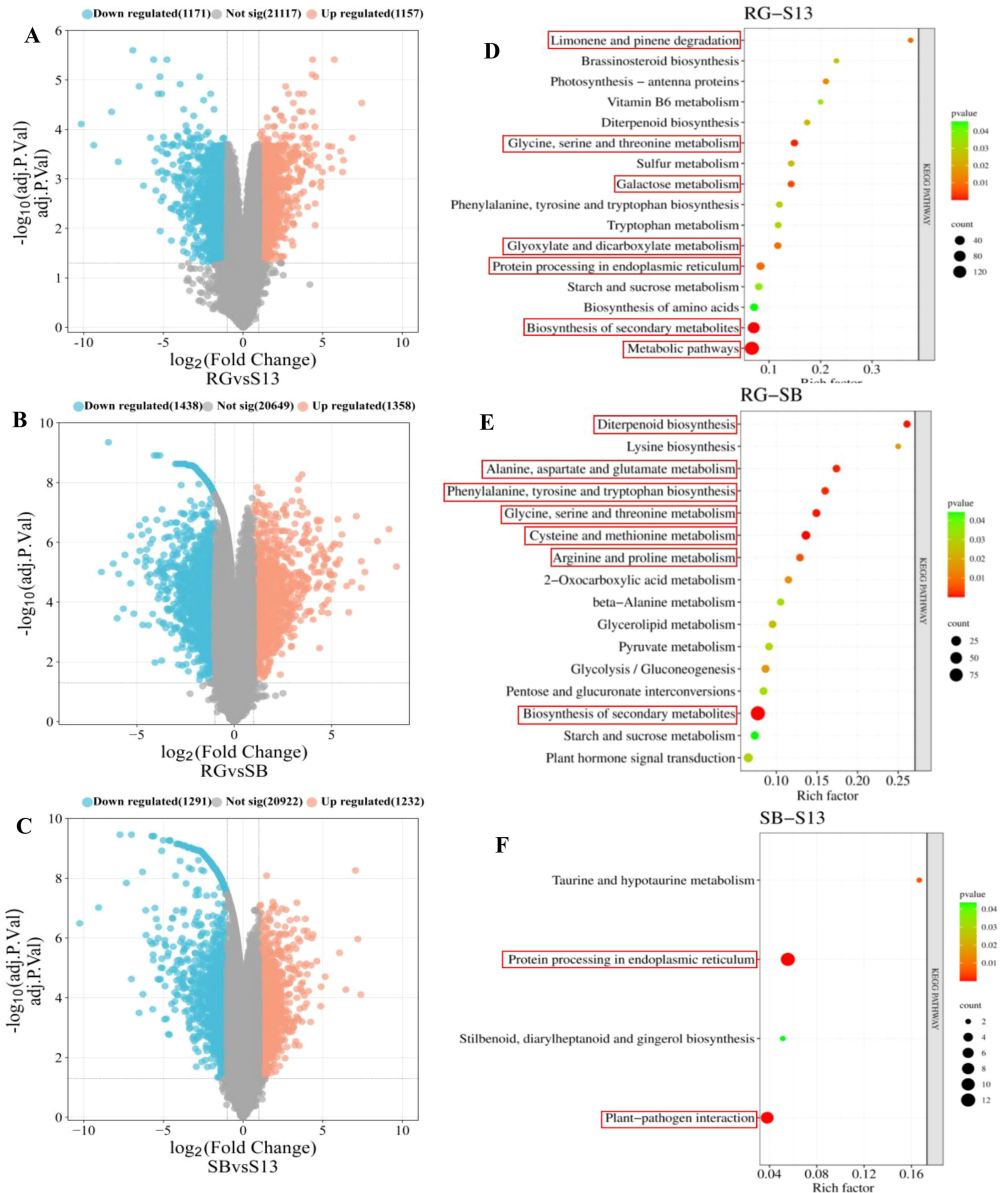
DEGs in three aroma types of grape varieties **(A–C)**. RG (‘Red Globe’), S13 (‘Shine 13’), SB (‘Summer Black’), and KEGG pathway analysis of DEGs. **(D)** RG (‘Red Globe’) vs S13 (‘Shine 13’); **(E)** RG vs SB (‘Summer Black’); **(F)** S13 vs SB; Count represents the DEG number. Gene ratios are calculated as the ratio of DEGs in each KEGG pathway compared with the total number of DEGs.

In this research, DEGs were calculated based on the FPKM value using R (v 4.2.3) with limma package, DEG analysis revealed upregulated and downregulated genes in three grape varieties of different aroma types (adj. *P*. Val < 0.05, |log_2_^FC^| ≥ 1) (**Figure 3**). A total of 2328 DEGs were identified in ‘RG’ (‘Red Globe’) and ‘S13’ (‘Shine 13’), of which 1157 and 1171 DEGs showed upregulated and downregulated expression levels, respectively (**Figure 3A**); A total of 2796 DEGs were found in ‘RG’ and ‘SB’ (‘Summer Black’), of which 1358 and 1438 DEGs exhibited upregulated and downregulated expression levels, respectively (**Figure 3B**); A total of 2523 DEGs were selected in ‘S13’ and ‘SB’, of which 1232 and 1291 DEGs showed upregulated and downregulated expression levels, respectively (**Figure 3C**).

To further investigate the functions of these DEGs concerning the biosynthesis of aromatic compounds, we performed KEGG pathway enrichment analysis. It was noticed that 7, 7, and 2 pathways that were significantly enriched (*P <*0.01) in ‘RG-S13’, ‘RG-SB’, and ‘SB-S13’, respectively; KEGG enrichment showed significant enrichment in limonene and pinene degradation, glycine, serine and threonine metabolism, galactose metabolism, glyoxylate and dicarboxylate metabolism, protein processing in endoplasmic reticulum and biosynthesis of secondary metabolites and metabolic pathways at ‘RG-S13’ (**Figure 3D**); The significantly enriched KEGG pathways included diterpenoid biosynthesis, alanine, aspartate and glutamate metabolism, phenylalanine, tyrosine and tryptophan biosynthesis, glycine, serine and threonine metabolism, cysteine and methionine metabolism, arginine and proline metabolism and biosynthesis of secondary metabolites at ‘RG-SB’ (**Figure 3E**). KEGG pathway enrichment analysis of the ‘SB-S13’ revealed enrichment in protein processing in endoplasmic reticulum and plant-pathogen interaction (**Figure 3F**). These significantly KEGG enrichment pathways mostly relate to the anabolism of amino acids, which were implicated in the anabolic and catabolic processes related to volatile compounds, including aldehydes, alcohols, esters, and methoxypyrazines.

### 3.8 Gene coexpression network analyses

**Figure 4:**
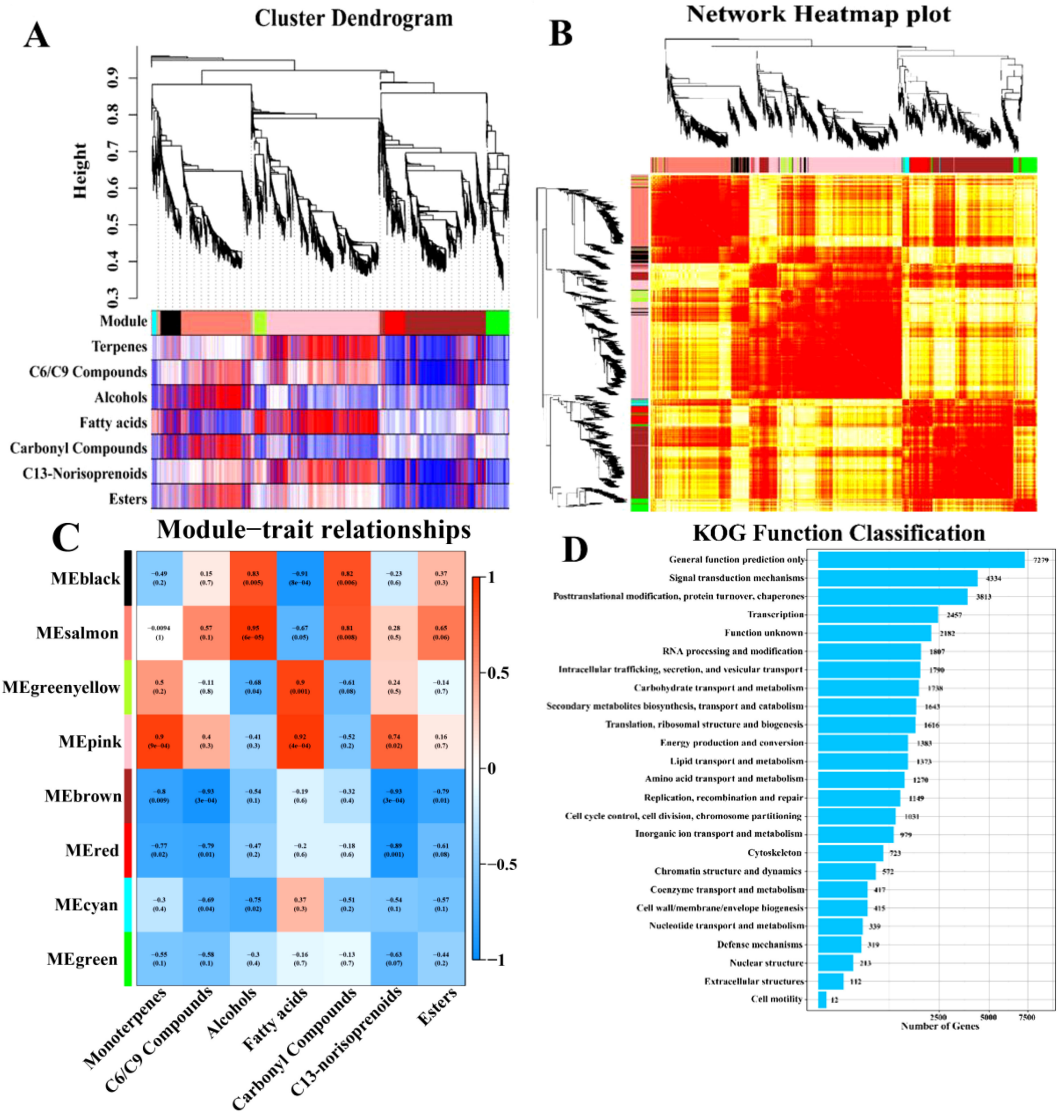
WGCNA and KOG function classification analysis in three aroma types of grape varieties. **(A)** Phylogenetic tree heatmap associated with volatile aroma compounds. **(B)** Gene co-expression network heatmap plot. **(C)** Heatmap of correlation between co-expressed gene modules and volatile aroma compounds traits. **(D)** KOG function classification in three aroma types of grape varieties.

With the acquisition of RNA expression data, and the aroma data of ‘RG’, ‘S13’, and ‘SB’ in the three different aroma types of grape berries, the gene co-expression network with weights was established using the optimal soft threshold, and 23,445 genes were selected for WGCNA analysis (**Figure 4B**). The genes were categorized into 8 distinct expression clusters, and a dendrogram of the gene clusters was constructed (**Figure 4A**). Based on the correlation results between modules and the concentration of aromas (**Figure 4C**), the MEpink (790 genes) and MEgreenyellow (110 genes) showed a significant correlation with monoterpenes, the MEblack (88 genes), MEsalmon (451 genes) and MEpink (790 genes) modules showed a significant correlation with C6/C9 compounds, MEblack (88 genes), MEsalmon (451 genes) modules were positively correlated with alcohols, MEgreenyellow (110 genes), MEpink (790 genes) modules were positively correlated with fatty acids, MEsalmon(451 genes), MEgreenyellow (110 genes) and MEpink (790 genes) modules were positively correlated with C13-norisoprenoids. MEblack (88 genes), MEsalmon (451 genes), and MEpink (790 genes) were positively correlated with esters.

The KOG function classification was performed on the RNA expression data (**Figure 4D**). There were 1373 and 1270 genes associated with lipid transport and metabolism and amino acid transport and metabolism, respectively. In addition, 1643 genes were related to secondary metabolites biosynthesis, transport and catabolism. These genes may regulate compounds related to aroma metabolism and catabolism.

### 3.9 Expression analysis of genes related to aroma biosynthesis in six types of grape cultivars

Monoterpenes and C13-norisoprenoids are predominantly produced through MEP and MVA pathways which occur in grapes plastids and cytosol, respectively (Wen et al., 2015). C6/C9 compounds, alcohols, carbonyl compounds, and esters are primarily biosynthesized in *β*-oxidation and lipoxygenase-hydroperoxide lyase (LOX-HPL) pathways (Lin et al., 2019; Schwab et al., 2008; Wang et al., 2001).

**Figure 5:**
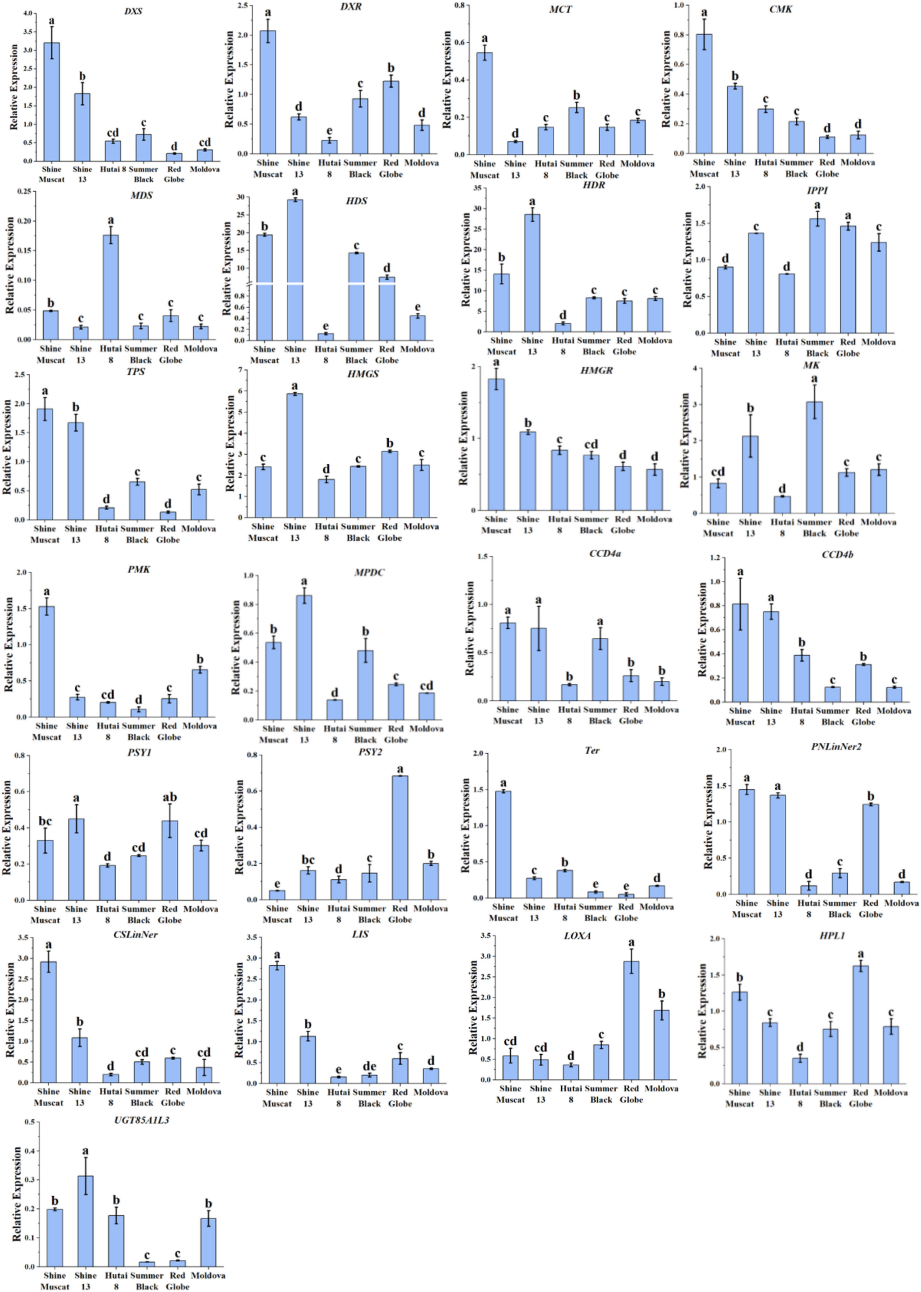
qRT-PCR analysis of the aroma related biosynthesis genes in six grape cultivars. Different letters in each graph indicated significant differences at *P* < 0.05.

The elevated expression of key terpenoid biosynthetic genes (*DXS, CMK, HDS, HDR, TPS, HMGR, CCD4a CCD4b, PNlinNer2, CSLinNer2* and *LIS*) in muscat-flavored cultivars (‘Shine Muscat’ and ‘Shine 13’) (**Figure 5**) strongly supports their role in monoterpene accumulation. Estévez et al. (2001) reported that *DXS* acts as the pivotal enzyme that initiates the first step in the MEP pathway. Furthermore, previous quantitative trait loci (QTL) analyses have indicated that *DXS* exhibited a strong correlation with the flavor profile of Muscat grape berries (Emanuelli et al., 2010), suggesting that genetic selection for *DXS* activity may drive cultivar-specific flavor differentiation. Overexpression of the *DXS1* gene in Nicotiana benthamiana leaves was demonstrated to enhance monoterpene biosynthesis, as reported by Wang et al. (2021). Notably, Yang et al. (2024) reported that relative expression levels of *CMK* and *HDR* were higher in muscat-type cultivars compared to neutral cultivars, which is consistent with our results. As a key rate-limiting enzyme in the MEP pathway, *HDR* regulates the synthesis of isoprenoids in plants (Banerjee and Sharkey, 2014). A previous study reported that *HDR* is the most expressed gene of the MEP pathway in ‘Sangiovese’ grape (D’Onofrio et al., 2018). Similarly, in this study, *HDR* also exhibited high expression levels in six grape cultivars. *TPSs* accelerate the concluding stage of free monoterpenes in the MEP pathway (Tholl, 2006). Until now, a total of 43 TPSs (terpene synthases) have been biochemically identified and characterized in grape berries (Lücker et al., 2004; Martin et al., 2010; Martin and Bohlmann, 2004). *TPS*, *PNlinNer2, CSLinNer*, and *LIS* belonged to *TPSs* gene family. *HMGR* is the key enzymes that catalyze the third step of the MVA pathway. A previous study indicated that the expression of *HMGR* genes was considerably elevated in Muscat grape varieties compared to strawberry-type cultivars (Zheng et al., 2021). Consistent with our results, the expression levels of *HMGR* in Muscat-type cultivars were significantly higher than in strawberry-type cultivars and neutral aromatic cultivars. The *CCD4* contributes to the creation of norisoprenoid aroma compounds and produces a range of water-soluble, low-threshold volatile compounds through the targeted cleavage of oxidized carotenoids (Zhong et al., 2020). The levels of *CCD4a*/*4b* expression were considerably high in Muscat-type cultivars. Additionally, the high abundance of *CCD4a*/*4b* suggests potential crosstalk between carotenoid cleavage and monoterpene modification pathways, which may diversify volatile profiles through secondary transformations. In line with our results, an earlier investigation found that the abundance of *CCD4* was more abundant in Muscat-type grape cultivars in comparison with those with a neutral aroma (Yang et al., 2024). These gene expression patterns correlated with the levels of terpene compounds, implying that these genes may play a predominant role in the variation of Muscat aroma among the six grape cultivars.

Among the five genes in *β*-oxidation and LOX-HPL pathways, *LOXA* and *HPL1* had the highest expression levels in ‘Red Globe’ (**Figure 5**). Our findings were in agreement with previous studies, which also reported that *LOXA* and *HPL1* exhibited high expression levels in neutral aromatic cultivars (Qian et al., 2016). Significantly, ‘Hutai 8’ had the highest expression levels of *AAT*, followed by the ‘Summer Black’ and ‘Shine 13’. Additionally, ‘Hutai 8’ exhibited the highest concentration of free and bound esters, which was consistent with the expression levels of *AAT*. It was proposed that *AAT* genes could substantially impact the enzyme activity of *AATs* in fruits, thereby influencing ester production (Balbontín et al., 2010; El-Sharkawy et al., 2005; Souleyre et al., 2005).

### 3.10 Correlations between the expression levels of genes involved in aroma biosynthesis pathways and the composition of aroma compounds across six grape cultivar types

**Figure 6:**
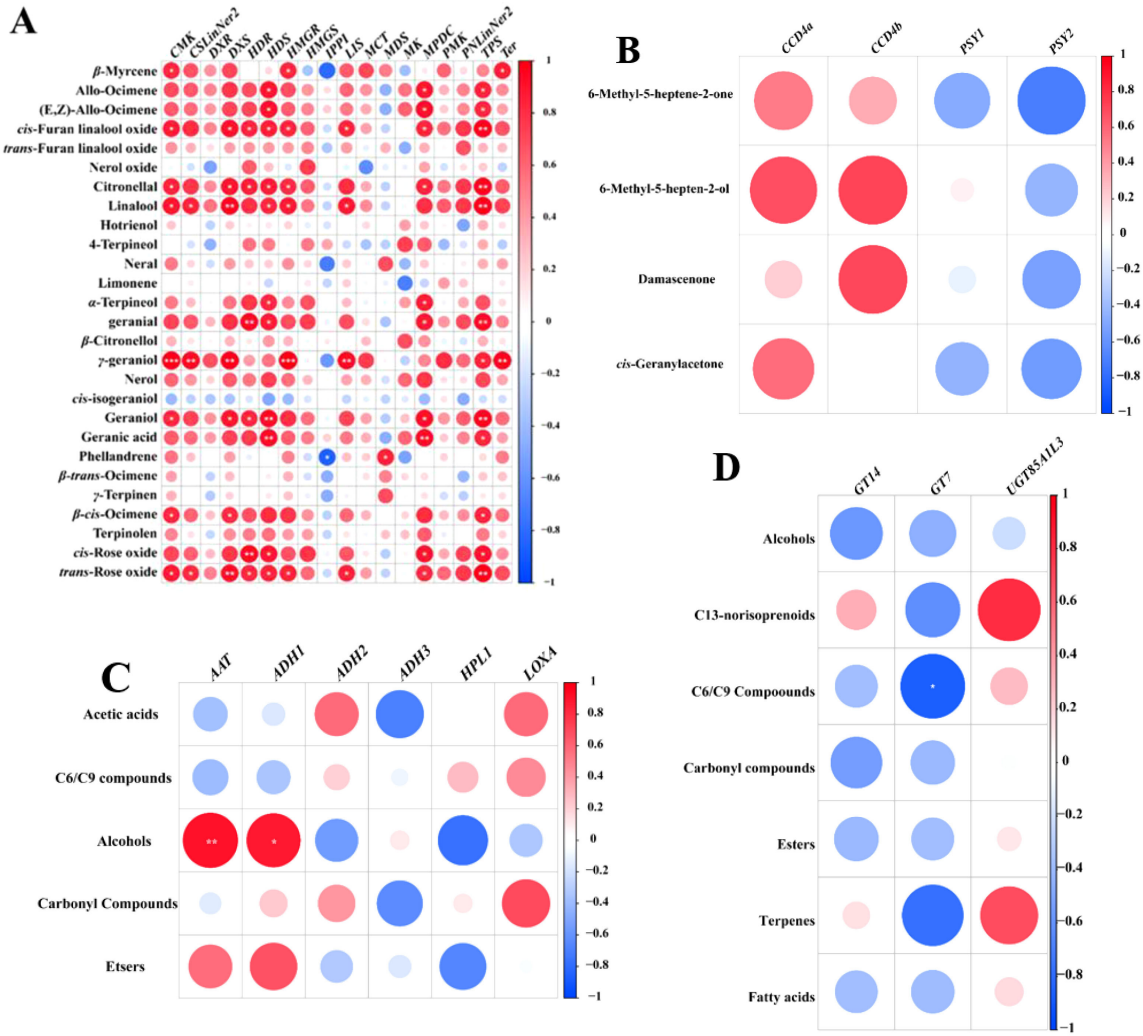
Correlations between the expression of aroma biosynthesis related genes and aroma compounds in six grape cultivars. **(A)** Correlation analysis of gene expression and monoterpene contents in MEP and MVA pathway; **(B)** Correlation analysis of gene expression and C13-norisoprenoids in MEP pathway; **(C)** Correlation analysis of gene expression and metabolite contents in LOX-HPL and *β*-oxidation pathway; **(D)** Correlation analysis of gene expression and bound aroma compounds. * indicates 0.01 < *P* < 0.05, ** indicates 0.001 < *P* < 0.01, *** indicates *P* ≤ 0.001.

To explore the link between aroma concentrations and gene expression across six grape cultivars, Pearson correlation analysis was performed, and visualized with a correlation heatmap (**Figure 6A**). It was observed that the concentrations of linalool, γ- geraniol, and trans-rose oxide were significantly positively correlated with the expression levels of *DXS.* The concentrations of linalool, geranial, and cis-rose oxide were markedly related to the expression levels of *HDR*. The concentration of geraniol and geranic acid was substantially correlated to the expression levels of *DXS*. Previous studies found the expression level of VvDXS shows a positive correlation with the accumulation of monoterpenes in grape berries (Costantini et al., 2017; Wang et al., 2021). The concentration of γ-geraniol was considerably correlated with the expression levels of *HMGR*, *LIS*, and *Ter*. Furthermore, *HMGR* is a rate-limiting enzyme in the MVA pathway, controlling the monoterpene synthesis, and facilitates the enzymatic conversion of HMG-CoA to MVA (Leng et al., 2023). Additionally, previous studies have demonstrated that overexpression of *HMGR* (*PtHMGR*) in Populus trichocarpa modulates the expression of genes associated with the MVA and MEP pathways and markedly enhances the synthesis of terpenoid compounds (Wei et al., 2019). The concentration of trans-furan linalool oxide, citronellal, linalool, geranial, geraniol, and trans-rose oxide showed a marked positive association with the expression levels of *TPS*. Similarly, several studies reported that *HDS, MVK, PNLinNer1*, and *DXS* showed strong correlations with monoterpenes accumulation (Yang et al., 2024; Ji et al., 2021; Zhou et al., 2022).

The expression levels of *ADH1* and *AAT* showed a marked positive association with the concentrations of alcohols and esters. (**Figure 6C**). Qian et al. (2019) observed that the genes (*LOXA* and *LOXO*) in the LOX pathway were positively related with their corresponding volatile esters. However, in this research, the expression levels of *HPL1* were negatively correlated with the concentrations of esters and alcohols, it may be due to the differences of varieties, environment and cultivation conditions. The expression levels of *CCD4a* and *CCD4b* were positively correlated with the concentration of C13-norisoprenoids, whereas the expression levels of *PSY1* and *PSY2* were negatively correlated with the concentration of C13-norisoprenoids (**Figure 6B**). *UGT85AIL3* expression correlated positively with bound C13-norisoprenoid and monoterpene concentrations, while GT7 expression showed a negative correlation with bound C6/C9 compound and monoterpene concentrations (**Figure 6D**). These discoveries align with the findings referred to in section 3.9 above.

## 4 Conclusions

This study provided comprehensive insights into the molecular and biochemical mechanisms underlying aroma diversity across six grape cultivars. There were 61, 61, 57, 56, 41, and 48 free aroma compounds as well as 59, 59, 55, 53, 48, and 46 bound aroma compounds were identified in ‘Shine Muscat’, ‘Shine 13’, ‘Hutai 8’, ‘Summer Black’, ‘Red Globe’ and ‘Moldova’, respectively. This reserach revealed distinct volatile profiles among the three aroma types, Muscat-type cultivars exhibited the highest levels of monoterpenes and C13-norisoprenoids, strawberry-type ‘Hutai 8’ was characterized by elevated ester content, while neutral aromatic cultivars showed dominance in C6/C9 and carbonyl compounds. PCA analysis further confirmed significant differences in aroma profiles, clustering the six cultivars into three distinct groups, aligning with their sensory classifications. Notably, there were 14, 13, 14, 12 12, and 11 volatiles with OAVs > 1 in six grape cultivars, respectively. Linalool, rose oxide, damascenone, and *cis*-geranylacetone were the main aroma compounds with OAVs >1 in Muscat-type grapes; ethyl butanoate was the main aroma compound with OAVs > 1 in strawberry-type grapes. RNA-sequencing and WGCNA analyses showed that 88 genes, 451 genes, 110 genes, and 790 genes had significantly positively correlated with alcohols, carbonyl compounds, fatty acids, and monoterpenes in three aroma types of grapes, respectively. Specifically, genes in the MEP pathway (*DXS*, *CMK*, *HDS*, *HDR*, *TPS*) and MVA pathway (*HMGR*) were highly expressed in Muscat-type cultivars, correlating strongly with monoterpenes accumulation. *LOXA* and *ADH2* expression peaked in ‘Red Globe’, while *AAT* dominated in ‘Hutai 8’, consistent with their respective volatile signatures. Crucially, correlation analyses highlighted functional linkages between gene expression and aroma compound synthesis. For instance, *CCD4a*/*b* expression positively correlated with C13-norisoprenoid levels, while *UGT85AIL3* was associated with bound monoterpenes and C13-norisoprenoids. Additionally, *AAT* and *ADH1* expression aligned with ester and alcohol biosynthesis, respectively.

An understanding of the aroma profiles metabolism and genetic mechanism of volatile compounds is particularly important for breeding new grape cultivars. These findings not only elucidate the genetic basis of cultivar-specific aroma formation but also offer a framework for targeted breeding strategies to enhance flavor complexity in grape berries.

## Data Availability

The datasets presented in this study can be found in online repositories. The names of the repository/repositories and accession number(s) can be found in the article/**Supplementary Material**.
