# Supplementary material for: Comprehensive aroma profiles and the underlying molecular mechanisms in six grape varieties with different flavors
Source: Front Plant Sci. 2025 Apr 28;16:1544593. doi: 10.3389/fpls.2025.1544593 (PMC12066448; doi:10.3389/fpls.2025.1544593)
Supplement: Supplementary file 2 [file Table2.docx]

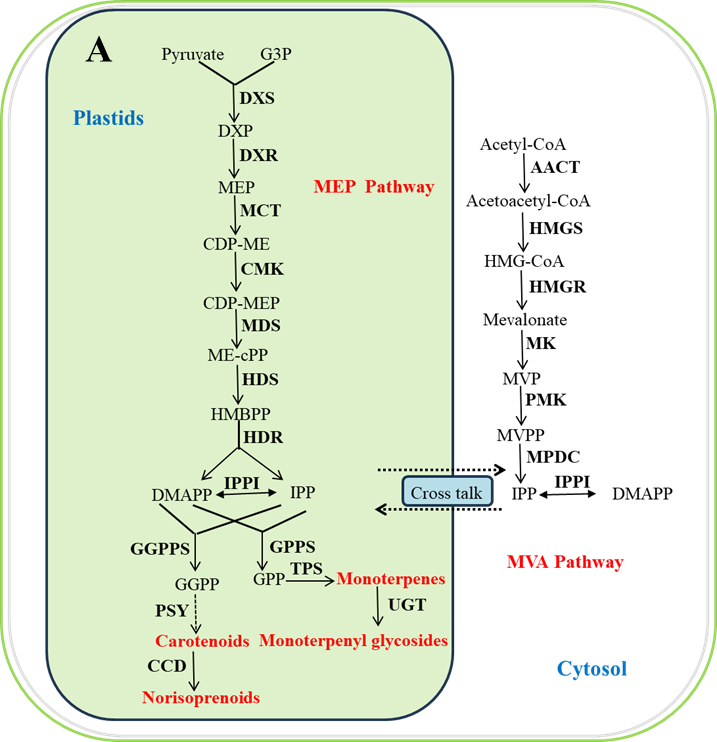

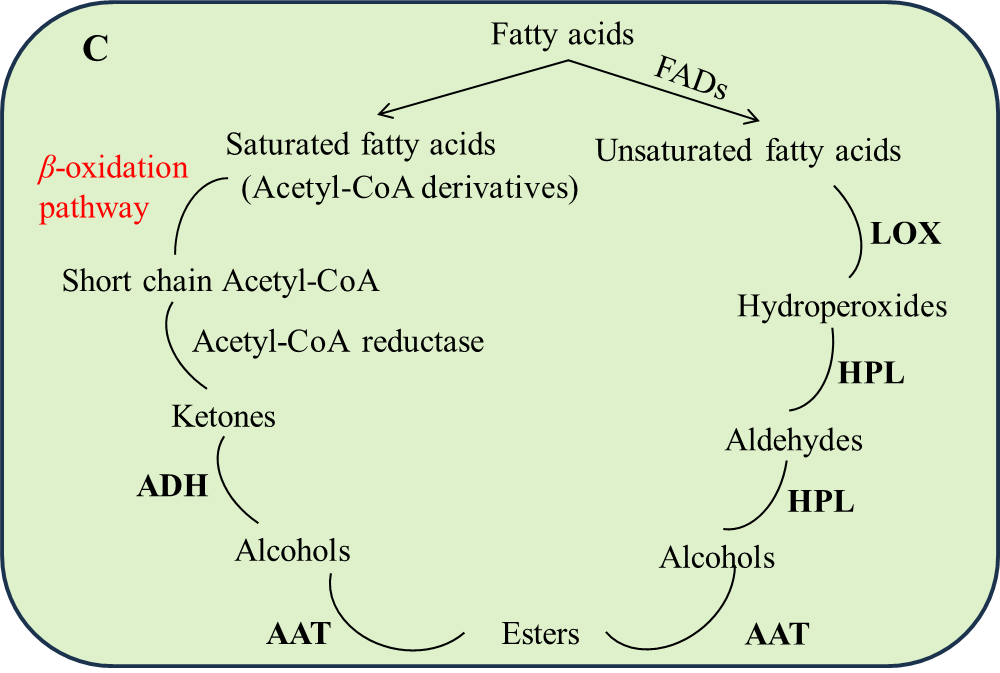

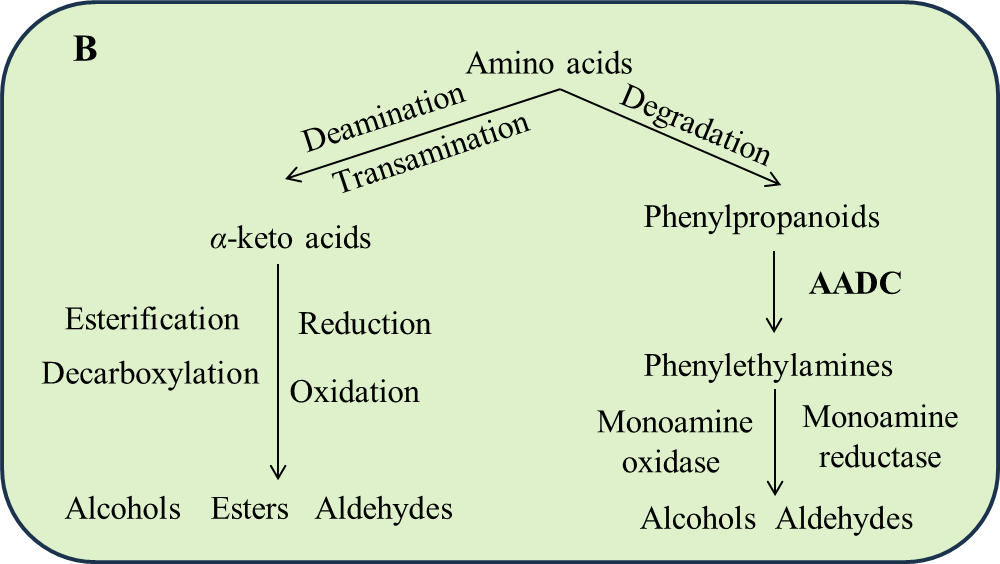


**Fig.S1** Schematic diagram of aroma compounds biosynthesis pathway in grape berries. A: The methylerythritol phosphate (MEP) and mevalonic acid (MVA) metabolic pathway. B: Amino acids biosynthesis metabolic pathway. C: Fatty acids biosynthesis metabolic pathway. AACT, acetyl-CoA C-acetyltransferase; HMGS, 3-hydroxy-3-methylglutarylCoA synthase; HMGR, 3-hydroxy-3-methylglutaryl-CoA reductase; MK, MVA kinase; PMK, phospho-MVA kinase; MPDC, diphospho-MVA decarboxylase; IPPI, isopentenyl diphosphate Δ-isomerase; DXS, 1-deoxy-D-xylulose 5-phosphate synthase; DXR, 1-deoxy-D-xylulose 5-phosphate reductoisomerase; MCT, 2-C-methyl-D-erythritol 4-phosphate cytidylyltransferase; CMK, 4-(cytidine 5 ́-diphospho)-2-Cmethyl-D-erythritol kinase; MDS, 2-C-methyl-D-erythritol 2,4-cyclodiphosphate synthase; HDS, 4-hydroxy-3-methylbut2-enyl-diphosphate synthase; HDR, 4-hydroxy-3-methylbut2-enyl diphosphate reductase; GPPS, geranyl diphosphate synthase; PSY, phytoene synthase; CCD, Carotenoid cleavage dioxygenase; TPS, terpene synthase; UGT UDP-glycosyltransferase; AADC*,* Aromatic L-amino acid decarboxylase; ADH, Alcohol dehydrogenase; LOX, Lipoxygenase; HPL, Hydroperoxide lyase; AAT, alcohol acyltransferase.
